# An Australian community jury to consider case‐finding for dementia: Differences between informed community preferences and general practice guidelines

**DOI:** 10.1111/hex.12871

**Published:** 2019-02-03

**Authors:** Rae Thomas, Rebecca Sims, Elaine Beller, Anna Mae Scott, Jenny Doust, David Le Couteur, Dimity Pond, Clement Loy, Cynthia Forlini, Paul Glasziou

**Affiliations:** ^1^ Centre for Research in Evidence‐Based Practice Bond University Gold Coast Queensland Australia; ^2^ Sydney Medical School The University of Sydney Sydney New South Wales Australia; ^3^ School of Medicine and Public Health The University of Newcastle Sydney New South Wales Australia; ^4^ Sydney School of Public Health The University of Sydney Sydney New South Wales Australia; ^5^ The Garvan Institute of Medical Research Sydney New South Wales Australia; ^6^ Sydney Health Ethics School of Public Health The University of Sydney Sydney New South Wales Australia

**Keywords:** citizen jury, community jury, dementia, general practice, primary care, public health

## Abstract

**Background:**

Case‐finding for dementia is practised by general practitioners (GPs) in Australia but without an awareness of community preferences. We explored the values and preferences of informed community members around case‐finding for dementia in Australian general practice.

**Design, setting and participants:**

A before and after, mixed‐methods study in Gold Coast, Australia, with ten community members aged 50‐70.

**Intervention:**

A 2‐day citizen/community jury. Participants were informed by experts about dementia, the potential harms and benefits of case‐finding, and ethical considerations.

**Primary and secondary outcomes:**

We asked participants, “Should the health system encourage GPs to practice ‘case‐finding’ of dementia in people older than 50?” Case‐finding was defined as a GP initiating testing for dementia when the patient is unaware of symptoms. We also assessed changes in participant comprehension/knowledge, attitudes towards dementia and participants’ own intentions to undergo case‐finding for dementia if it were suggested.

**Results:**

Participants voted unanimously against case‐finding for dementia, citing a lack of effective treatments, potential for harm to patients and potential financial incentives. However, they recognized that case‐finding was currently practised by Australian GPs and recommended specific changes to the guidelines. Participants increased their comprehension/knowledge of dementia, their attitude towards case‐finding became less positive, and their intentions to be tested themselves decreased.

**Conclusion:**

Once informed, community jury participants did not agree case‐finding for dementia should be conducted by GPs. Yet their personal intentions to accept case‐finding varied. If case‐finding for dementia is recommended in the guidelines, then shared decision making is essential.

## 1 INTRODUCTION

Early diagnosis of dementia is a challenging issue for primary care physicians, largely due to concerns arising from the fragile balance of harms and benefits,^1^, ^2^, ^3^ the imprecision of some assessment tools^4^, ^5^ psychological distress, reduced quality of life^6^ and financial burden.^3^ Multiple countries including Australia, the UK, the USA and Canada have guidelines that consider “early identification” of dementia but these countries do not support screening for dementia.^5^, ^7^, ^8^, ^9^

Screening for dementia occurs in people who are asymptomatic (eg, a certain age, individuals “at risk”).^10^ Case‐finding occurs when clinicians are triggered to explore a dementia assessment because of cues from an individual's symptoms or behaviour, in combination with pre‐existing knowledge and clinical judgement, but the individual is unaware of signs of dementia^11^ and had presented to the health clinic for another reason. In contrast, “timely diagnosis” occurs when patients or carers present to physicians concerned their symptoms may be dementia and request an assessment^3^ or when a symptoms adversely affect the person or those close to them.^12^ Globally, increased awareness of dementia in the public and media has resulted in many advocating for early diagnosis.^1^, ^13^ There are two interrelated issues that arise from this: first, how early is early—at what time point should dementia be identified; and second, what approach is best for “early identification”—screening, case‐finding or timely diagnosis?

Recently, the Royal Australian College of General Practitioners (RACGP) updated their practice guidelines^14^ to encourage general practitioners (GPs) to practise “case‐finding” for dementia in people over 65. The recommendation is for GPs to be “alert to the signs and symptoms of dementia” and practise case‐finding by asking “how is your memory?” and obtaining information from reliable others over several appointments. Case‐finding can be considered controversial, as on the one hand, it may allow for more timely planning and identification of the disease, but on the other, it may also result in a potentially devastating diagnosis with few effective treatment options, turning a seemingly well person into a sick person for an extended period of time.

Because of the fragile balance between potential harms and benefits of case‐finding, we need to explore community values and preferences before case‐finding for dementia becomes an agreed practice. We conducted a citizen/community jury (CJ) using methods based on those described by the Jefferson Centre^15^ to consider the informed community perspective about whether GPs should practice case‐finding for dementia. CJs are a form of deliberative democracy used to explore community perspectives on important but controversial topics.^15^ CJ participants are recruited from the general population or the target population for the condition being studied and deliberate on questions requiring an ethically sensitive or values‐based decision.^16^ CJs aim to elicit an *informed* community perspective on difficult topics where the values and preferences of community members enhance policy decisions.^17^ CJ members are provided with expert presentations and opportunities to question the experts, engage in both facilitated and private deliberation, and are asked to form a consensus or majority “verdict” on the topic question.^15^ CJs have been used successfully in research to elicit informed perspectives for several health policy issues, for example screening mammography,^18^, ^19^ screening for prostate cancer,^20^ quantifying health preferences^21^ and more broadly in local governments.^22^, ^23^

Our primary outcome was community juror recommendation for the question, “Should the health system encourage GPs to practice “case‐finding” of dementia in people older than 50?” We deliberately lowered the age range from the guidelines to over 50 years to reflect both Australian public experiences of other health practices such as cancer screening programmes (eg, government sponsored bowel and breast cancer screening commence at age 50 in Australia), and bone density checks, etc. that heighten awareness of individual health concerns, and to reflect that younger‐onset dementia (although rare) is increasingly recognized as a potential problem confronting practitioners.^24^ We also assessed changes in participant comprehension/knowledge of dementia, attitudes towards dementia, whether they had engaged in an informed decision, and explored consistency of participant's own intentions to test for dementia.

## 2 METHOD

### 2.1 Participants

We recruited from the age group most affected by the question^25^—50‐ to 70‐year‐olds. We recruited individuals from the Gold Coast Region (Australia) with no (self‐reported) previous diagnosis of dementia, Alzheimer's disease (AD) or mild cognitive impairment (MCI). CJs aim to include participants directly affected by the CJ question. For this study, we deliberately recruited participants who were in the age bracket most likely to be impacted by GPs case‐finding for dementia and individuals with no dementia diagnosis. We excluded participants with immediate family members (parents, partners, in‐laws, children or siblings) diagnosed with dementia, AD or MCI, individuals caring for someone with these conditions, and individuals actively taking cognitive‐enhancing medications.

Participants were recruited by the Social Research Centre (Central Queensland University) using a randomly selected landline‐based sample with quotas to ensure gender and education balance. Once recruited, participants were contacted by the research team, provided further information, asked for verbal consent and given details for their attendance. Participants received two $100 gift cards to reimburse their time. Bond University Human Research Ethics Committee (#15810) provided ethics approval.

### 2.2 Presenting experts

We invited the four experts to present to the CJ based on their clinical expertise and their publicly stated positions towards case‐finding for dementia on committees, in published documents or both. Each has clinical or research experience with patients with AD and dementia. The scientific expert is a clinical epidemiologist and cognitive neurologist, and the ethics expert researches neuroethical issues in the ageing population. The expert presenting the potential negatives for case‐finding for dementia works in geriatric medicine with expertise in ageing and AD. The expert presenting the potential benefits of case‐finding for dementia is a GP with expensive experience working with patients with dementia and chaired the RACGP practice guidelines on dementia. All experts had access to each other's presentation. Presenters, their topics and access to their presentations via URLs are provided in Box 1. After completion of the CJ, expert presenters contributed to the writing of the manuscript and are named as co‐authors. No reimbursement (financial or otherwise) was provided to the experts.

Box 1 **Expert presentations and download links**

1

1. What is dementia, how is it diagnosed, and what are the treatment options(Clement Loy) https://youtu.be/ssFmga7p39Q
2. The ethics of case‐finding for dementia(Cynthia Forlini) https://youtu.be/iz-3hWiw5Jw
3. The potential harms of case‐finding for dementia(David Le Couteur) https://youtu.be/l1tK8NFfhjw
4. The potential benefits of case‐finding for dementia(Dimity Pond) https://youtu.be/lqgn8VHO5CI

### 2.3 Materials provided to CJ participants

Participants were provided with biographies of the experts, the schedule of the weekend, and during private deliberations, a copy of the relevant section of the RACGP Redbook practice guidelines for case‐finding of dementia.^14^ In addition, a printout of the definitions used in the CJ for screening, case‐finding and diagnosis was also provided. The definition for case‐finding used in the CJ was “*Case‐finding*—a patient may incidentally complain about a problem (ie, it is not the presenting problem) that triggers suspicion on the GPs behalf and so is tested for dementia (eg, complains of losing words three times this week, forgetting keys etc.).” These patients are unaware this incidental disclosure may indicate signs of dementia. See Appendix S1 for all definitions.

### 2.4 Patient and public involvement

We did not involve patients in the design or recruitment of this CJ. However, the content and structure of the CJ were designed and implemented by considering the feedback and suggestions from community jurors who participated in previous juries this team had conducted. Community jurors for the present CJ are acknowledged and thanked in the acknowledgement section collectively, as identifying them individually by name would risk compromising their anonymity. CJ participants were asked whether they were interested in receiving the publication detailing the results of the CJ, and those who explicitly consented will be provided with the published version of the article.

### 2.5 Procedure

The CJ was conducted over two weekend days, 18‐19 March 2017, at Bond University (see Table 1 for schedule). All sessions except for the final deliberation were facilitated by a research team member RT (a psychologist) to ensure equal participation, record questions and note participant concerns. Throughout the 2 days, except during the final confidential deliberation, two observers (RS and AMS) took contemporaneous notes on participant comments, affect and participation to support the facilitator. So as not to lead or bias the jurors towards a specific recommendation, no one outside of the jury group was present during private deliberations.

**Table 1:** Community jury schedule

Saturday		
9.00‐9.30	Overview of community jury	Rae Thomas
9.30‐10.00	What is dementia, how is it diagnosed what are the treatment options	Clement Loy
10.00‐10.30	Questions	
10.30‐11 am	MORNING TEA	
11.00‐11.30	The ethics of case finding for dementia	Cynthia Forlini
11.30‐12.00	Questions	
12.00‐12.30	LUNCH	
12.30‐1.00	The potential harms of case finding for dementia	David Le Couteur
1.00‐1.30	Questions	
1.30‐2.00	The potential benefits of case finding for dementia	Dimity Pond
2.00‐2.30	Questions	
Flexible timing in response to Juror needs	Jury deliberations Stage 1	Rae Thomas
	AFTERNOON TEA	
	Questions and Close	
Sunday		
9.00‐9.30	Reconnect and Debrief	Rae Thomas
9.30‐10.30	Further questions and deliberations	Rae Thomas (or private if jurors ready)
Flexible timing in response to Juror needs	MORNING TEA	
	Deliberations until consensus or impasse	
	LUNCH	
	Deliver Verdict	
	Debrief, Process discussion and close	

On Saturday, participants provided written consent and completed the pre‐CJ (baseline) survey. Experts with clinical and research expertise in the areas of cognitive impairment and dementia, geriatric medicine, epidemiology and ethics spoke about specific information about dementia, ethical considerations regarding case‐finding, and the perceived benefits and harms of case‐finding for dementia in general practice. Each expert presented a 20‐minute voice‐over slide presentation (see Box 1 for details) followed by a telephone question and answer session. Participants were provided with presenters’ biographies and handouts of their presentations.

On Sunday, participants debriefed, discussed overnight reflections and were provided the opportunity to recontact the experts via telephone for further information and clarification. Participants then deliberated in private on the primary question and were able to ask for clarification on any matter during this time. They then presented their decision to the facilitator and researchers.

### 2.6 Measures

It is important to ascertain that CJ participants made an “informed decision” when providing their recommendations. This requires adequate comprehension of the topic and a consistency between their personal attitudes towards the topic and their personal intentions.^26^

Information questions are used in CJs to assess participant comprehension of information presented to them by the experts during the CJ. Therefore, questions and answers are developed from the expert presentations and are reflective of the information provided and are not meant to be reflective of higher clinical or research knowledge. Ten comprehension questions were developed from information provided during the expert presentations (seven true/false conceptual items and three multiple choice numerical items). Post‐CJ adequate comprehension was defined a priori as 50% correct.^27^

Attitudes towards case‐finding for dementia were assessed using five items on a 7‐point scale with the higher number suggesting more positive attitudes.^26^, ^27^, ^28^ A positive attitude was defined as scores ≥28/35.^27^

We measured future intention to undergo case‐finding for dementia if suggested by a GP using a 7‐point scale ranging from 1 (definitely not) to 7 (definitely will). Scores between 5‐7 were classified as positive intentions, and scores between 1‐3 and 4 (unsure) as negative intentions. To explore the time and information provision required for an individual to achieve consistent responses, we asked participants this same question on nine occasions: baseline; after each expert presentation; at the end of day 1; at the start of day 2; after deliberation; and at the end of day 2.

Informed choice was defined as adequate relevant knowledge and a consistency between individual attitudes and intentions.^26^, ^27^ The post‐CJ survey is available in the Appendix S1.

### 2.7 Statistical analyses

The CJ proceedings were audio‐recorded and transcribed. Participants’ recommendations on the primary question were also recorded on a whiteboard, corrected by participants and participant notes were also provided. Transcripts were analysed qualitatively to identify reasons for juror recommendations. We analysed comprehension/knowledge, attitudes and intentions in a before and after study design. Paired pre‐ to post‐CJ differences for continuous outcomes were examined using Wilcoxon signed rank tests. All data were analysed in SPSS Statistics 23 (IBM Corp., Armonk, NY, USA).

## 3 RESULTS

Of the 14 participants recruited, 12 were available for the weekend and agreed to participate in the study. Of these, one withdrew prior to day 1 (male aged: 60‐70 years), and one did not attend for unknown reasons (female aged: 60‐70 years). Ten participants attended and completed the CJ. The average age of participants was 62 years (SD = 6.9; median 62.5 years, IQR = 12.25), and there was an even gender split. Education levels were mixed. Nine participants indicated they had not been tested for dementia and one was unsure (Table 2).

**Table 2:** Participant demographics (N = 10)

Age	
Mean (SD)	62 (6.9)
Median (IQR)	62.5 (12.25)
Male (n)/female (n)	5/5
Previous MCI/dementia test (n)	
Yes	0
No	9
Don't know	1
Education (n)	
Some high school	3
Grade 12	4
Some university/TAFE	2
University postgraduate	1

MCI, mild cognitive impairment; TAFE, technical and further education institutions.

### 3.1 Community jury recommendation

Community jury participants engaged with each presentation and asked questions of each presenter immediately following their presentation. On the morning of day 2, CJ participants also asked further clarifying questions to speakers 1, 3 and 4 (See Table 1 for speaker details). After the deliberations on day 2, participants voted unanimously (10/10) against the jury charge: “Should the health system encourage GPs to practice case‐finding of dementia in people older than 50?” Reasons included the following: lack of effective treatments for dementia (ie, cure), case‐finding may occur too early in the course of the disease, the impact case‐finding might have on an individual's mental health, the role of the GP and the potential financial incentives for case‐finding (Box 2). The wording of the charge challenged the participants. They believed the age of case‐finding in the charge at 50 years was “too young” (J2) and thought the word “encourage” might translate to financial incentives.

Despite a unanimous “no” verdict, participants recognized that because guidelines for case‐finding of dementia were outlined in the RACGP Clinical Guidelines for Preventive Activities in General Practice, GPs were currently practicing case‐finding for dementia. Participants requested and were given a copy of the relevant section in the RACGP Clinical Guidelines and they made alterations they considered would “stop it [case‐finding for dementia] from happening in a harmful way” (J7). (J7 was nominated by the participants pre‐deliberation as the fore‐person and spokesperson.) J7 “So given that it's here to stay, we'd like to adjust these [RACGP] guidelines.” Importantly, despite the current guidelines suggesting case‐finding occur in people over 65, and participants believing the age of 50 in the jury “charge” was too young, the jurors recommended removing the age criterion from the guidelines. They reasoned that as dementia did occur in younger ages (although rare), by removing age caveats “all individuals would have equal access and equitable treatment regardless of age.” The participants’ recommended changes to the section referencing case‐finding for dementia in the RACGP Clinical Guidelines are provided without edit in the Appendix S1.

Box 2 **Justifications for jury decision**

**No effective treatment**

Juror 5: I think that until there is a definite chance of stopping or fixing the problem, it would create a far greater negative outcome than a positive one.

Juror 1: It was a surprise to me that I didn't realise there was actually nothing that could be done to help anybody with it.

**Case‐finding too early in the course of the disease**

Juror 5: You know, we're getting told very early when it's going to be 10 years before it appears, that would be 10—for a lot of people, that would be 10 years of worry.

Juror 6: I look at it this way, that the diagnosis stage is still early enough for planning.

**Role of the GP**

Juror 1: GPs overstepping role “Unless the patient specifically has a concern that they speak to their GP about, then I don't think the GP should step in. I think it's for testing, screening, whatever, that is something that is entirely up to the patient.”

Juror 1: I just think, what gives a GP a right to play god?

Juror 2: When somebody mentions dementia or Alzheimer's to somebody, you are placing fear into their mind….I don't think it is the doctor's right to set somebody up with that fear.

**Mental health**

Juror 10: I see that to be diagnosed and told that you are destined to become a person with dementia, will be devastating for anyone. For those patients who are misdiagnosed and caused unnecessary fear and indignity, it would be far worse.

Juror 5: I was involved a lot in the AIDS thing way back and there were people hearing they had it and going out and killing themselves, like that, you know, just the shock. So the same thing could apply with this. It's a death sentence in a way.

Juror 2: The stress and anxiety of people that might get diagnosed or misdiagnosed just outweighs the positives that might be.

Juror 1: They just don't know what effect mentally that's going to have on that person.

**Potential incentivisation**

Juror 2: …encouraging GPs would just encourage kickbacks and overdiagnosis because people, like people are, they want to profit.

Juror 4: I had a doctor telling me once about try these things and telling me about the holiday he had because of the incentive.

**Less frequently expressed concerns**

Juror 6: Has any one of us considered the cost factor on the whole community? Because all the screening and referrals to specialists and counsellors and ‐ it must be huge and basically for nothing.

Juror 2: Your medical insurance would dump you like a brick.

In addition, to specific guideline changes, the participants suggested potential solutions (Box 3). For example, although the participants thought they knew about dementia from media and public discourse, they were surprised to learn that there are currently no effective treatments and that prevention rests upon modifiable risk factors that may decrease risk of dementia but not eliminate it. Therefore, participants believed the public were not fully cognisant of information about dementia required to make an informed health decision. Participants recommended a public awareness campaign. Finally, in response to concerns about financial incentives for case‐finding for dementia, the participants suggested that any potential incentives be invested into research to address prevention and management of dementia.

Box 3 **Potential solutions suggested by Jurors**

**Public awareness**

Juror 8: there needs to be more education so people can sort of make an informed choice of whether they want to go and talk to their GP about it.

Juror 7: we'd like to add in education and awareness programs…… [about] dementia, the signs, the symptoms and the processes and treatments and supportive systems…

Juror 5: also lifestyle education…

Juror 7: at an early age.

**Reallocation of any potential incentives**

Juror 2: Wouldn't it be nice, if the government, instead of giving kickbacks to doctors or pharmaceutical companies ……. if they all [the government] put their money in more research to cure the damn thing in the first place?

Juror 6: I think it would be good if legislation, government legislation was passed that all incentives, from wherever they come, should be diverted from the doctor to a research facility.

Juror 7: ……… because research funding is so scarce, any incentive a doctor is given to refer to a memory clinic to any other centre, that incentive does not go into the doctor's pocket, it goes into a funding body for research into dementia.

### 3.2 Comprehension, attitudes and intention to test

At pre‐CJ, participant comprehension/knowledge about dementia was good with eight participants scoring 6 or 7/10 correct and two participants scoring 4/10 correct. Overall, comprehension scores significantly increased from pre‐ to post‐CJ (median: 6, IQR: 6‐6 vs median: 7, IQR: 7‐8, *P* = 0.004; Table 3). At post‐CJ, all participants had adequate comprehension based on presentation information with nine participants achieving 7 or 8/10 correct and one participant scoring 5/10.

**Table 3:** Differences in comprehension/knowledge, attitudes and intentions pre‐ to post‐community jury

	N	Pre‐CJ			Post‐CJ			Wilcoxon *P*‐value
		Median	Q1	Q3	Median	Q1	Q3	
Comprehension/knowledge total (/10)	10	6	6	6	7	7	8	0.004
Attitudes total (/35)	9	30	22	34	12	6	20	0.01
Intention to test (/7)	10	7	6	7	2	1	6	0.01

Before the CJ, participants’ attitudes towards case‐finding for dementia were mixed with four participants reporting an overall negative (score of <28) and five participants an overall positive attitude (≥28, median = 30, IQR = 22‐34). Data were missing for one participant. However, after the CJ, participants reported significantly less favourable attitudes with only two participants maintaining overall positive attitudes towards case‐finding (median = 12, IQR = 6‐20, *P *=* *0.01).

**Figure 1:**
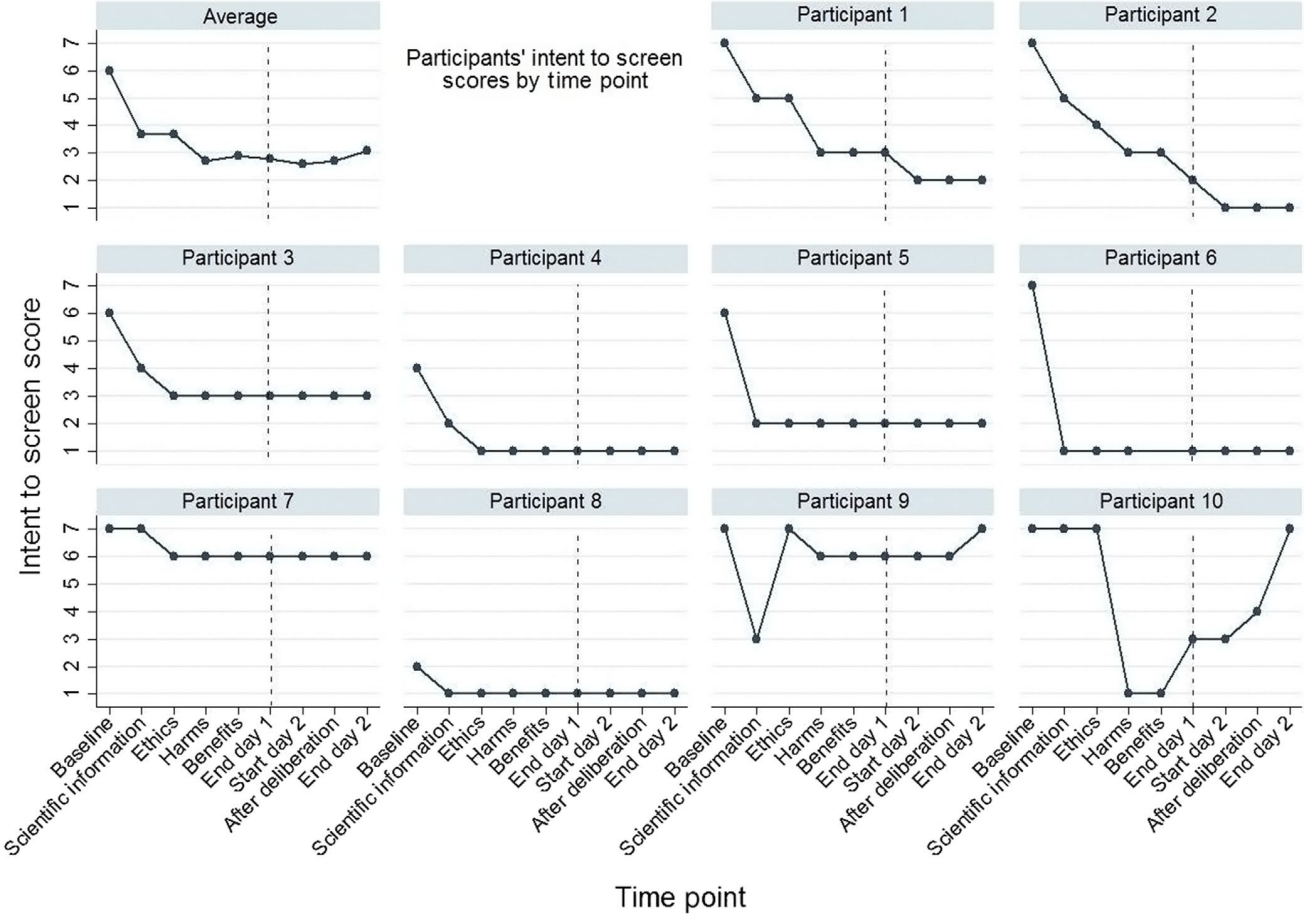
Comparison of individual intention to test scores over the community jury weekend

Pre‐CJ, most participants reported positive intentions to undergo case‐finding for dementia should it be suggested (8/10; Figure 1). However, only three participants thought this post‐CJ. This was a statistically significant decline in the overall intention to test score (median = 7, IQR = 6‐7, vs median = 2, IQR = 1‐6, *P *=* *0.01).

### 3.3 Informed decision

Using the algorithm for informed decision making (≥50% comprehension questions correct and congruence between attitude and intentions to test), nine participants made an informed decision post‐CJ. The remaining participant scored negatively on their attitude towards case‐finding (score of 20) but indicated they “definitely will” (score of 7) undergo case‐finding for dementia if offered.

### 3.4 Consistency in decision making

Seven participants decreased their individual intentions to undergo case‐finding after the first expert presentation. After this change, most (6/10) maintained their individual intentions (positive, negative or unsure) to test for dementia after either the scientific or ethics presentations (Figure 1). Two participants continued to decrease their intentions to test until the start of the second day after which they remained consistent. Two other participants changed their individual intentions up until the end of the assessment period.

## 4 DISCUSSION

Our informed community members did not believe Australian GPs should practice case‐finding for dementia. Their reasonings included the futility of being diagnosed in the absence of symptoms of impairment when there was little evidence of effective prevention or treatment strategies and that case‐finding (compared with timely diagnosis) had the potential to evoke unnecessary worry. In addition, after the CJ, participants on average increased their comprehension of dementia (according to information presented to them), decreased their positive attitudes towards case‐finding and their own intention to undergo case‐finding for dementia should it be suggested, and made an informed decision regarding the CJ question.

Research supports their position. A recent systematic review on the benefits and challenges of timely diagnosis of AD^3^ reported that some challenges/harms faced by potential patients included fear, anxiety, worry and stigma. Unlike our definition of case‐finding, these reported challenges were within the context of people presenting specifically with concerns about their cognitions. In another study, individuals diagnosed with MCI compared with individuals with normal cognitive functioning reported a reduced quality of life, significantly more depression and stress.^6^ Additionally, the same study reported that individuals unaware of their diagnosis of either MCI or AD reported greater quality of life and better well‐being than those aware of the diagnosis,^6^ suggesting that regardless of symptoms, the diagnostic label itself was harmful to some.

Despite their opposition, the community members recognized case‐finding was promoted in the RACGP Clinical Guidelines for people over 65 years.^14^ So, CJ participants suggested changes to the guidelines including wide‐scale public education regarding diagnostic, prognostic and treatment uncertainty; clinician education on discussing this uncertainty; and concerns about any potential financial incentives by explicitly prohibiting these, and instead redirecting any monies towards preventive and treatment research. Despite concern regarding the low age of case‐finding posed in our CJ question, participants suggested eliminating the age criterion to reflect the rare but important possibility of early age onset.

The study has several strengths. This was the first CJ we are aware of to explore community values and preferences of case‐finding for dementia. CJs provide participants with expert information and the ability to question the experts, thus capturing participants’ *informed* views and preferences. This contrasts with other forums that garner public opinion which lack the information provision element, such as focus groups and population surveys. For example, public views on screening for prostate cancer are generally positive. When we conducted a CJ on this topic, pre‐CJ screening intentions and attitudes were positive (as would be expected)^20^; however, post‐CJ this position was reversed. As is usual practice, in this CJ we selected our participants following CJ practices of randomly recruiting from the “affected public.”^16^ As CJs recruit participants who are potentially affected by the question^16^ (case‐finding), we deliberately excluded carers and individuals diagnosed with MCI or dementia. Our participants therefore represent the authentic experiences of service users with no vested interest in the topic.^16^ We acknowledge the jury decision may have been different should other members of the public have been included. For example, previous research reported that 92% of individuals attending a memory clinic to assess their cognitive functioning wanted to know the outcome of their assessment.^29^ However, these people had already consented to testing so were unlike participants affected by our question. Although review papers report “most people want to know,”^30^ participants in this CJ (the affected public for case‐finding) were mixed in their individual intentions to undergo testing yet unanimously against its common practice. Participant comments suggest broad public awareness of dementia but a more limited understanding of issues related to prognosis and treatment. Finally, to aid reproduction and transparency, the reporting of the CJ complies with the CJCheck reporting protocol^31^ and all presentations are available for viewing.

However, there are also limitations. Lowering the age range in the RACGP guidelines from 65 to 50 years, which reflects current diagnostic concerns of younger‐onset dementia^24^ and aligns with other screening health practices in Australia, may be a limitation because it does not reflect current guideline recommendations. However, although the jurors initially thought this age “too young” their final recommendations to the RACGP included eliminating the age criterion altogether. Their justification for doing so was to acknowledge the rare occasions of younger‐onset dementia and to increase “equal access and equitable treatment regardless of age.” This is an example of where community juror recommendations would need to be viewed by epidemiologists before potential implementation. Removing age criterion for case‐finding for dementia would significantly lower the positive predictive value of diagnosis rates because the prevalence of dementia in young age groups is very low.

By design, CJs are small^18^, ^19^, ^20^, ^21^ and this is often a criticism. CJ participants are not suggested to represent the larger population. They should be selected randomly with quotas of important characteristics relevant to the topic (eg, gender, age range). If this CJ was repeated with different participants, with different values and preferences, the recommendations may differ from those described here. Outcomes of CJs may not be able to be replicated but methods are reproducible. Recently, two CJs on antimicrobial stewardship were conducted in different settings with similar results suggesting once informed, participants (in different regions but recruited for characteristics relevant to the juror question) may make similar recommendations.^32^ Also, careful consideration is taken to select experts with known clinical and research expertise and with differences of opinion (for and against case‐finding). They had access to each other's presentations for transparency and potential comments and research presented to support their claims were referenced. However, CJ participants can only be “informed” from the information provided by these experts. If different experts had been selected, different information may have been provided. CJs are only used for controversial topics; in this arena universal truths are rare.

It is not suggested that Australian GP guidelines change because of the CJ recommendations; indeed, the recommendation to remove the age requirement would require considerations of the sensitivity and specificity of any tests with a different age cut‐off which is beyond the information provided to the jurors. But this study does highlight some important implications for guideline developers and clinicians.

Research has demonstrated guideline development groups and panels that decide new definitions of disease or diagnostic practices often comprise panel members with financial ties to pharmaceutical companies, and/or emotional and academic vested interests.^33^ Missing from these groups are community voices, values and preferences. CJs provide a mechanism to elicit informed community values and preferences which can help inform guideline and panel groups. For clinicians, the assumption that most individuals would want to know about a diagnosis of dementia, when made before symptoms are known to the patient, is contestable. When informed about the potential harms and benefits of case‐finding for dementia, community members were unanimously against a universal service and mixed in their individual health‐care decisions. Australian GPs should carefully consider case‐finding for dementia in their practice given the lack of effective treatments and the potential to add years of stress and uncertainty to patients’ lives. Shared decision making^34^ is essential when views are so mixed.

## CONFLICT OF INTERESTS

None.
