# Supplementary material for: An Australian community jury to consider case‐finding for dementia: Differences between informed community preferences and general practice guidelines
Source: Health Expect. 2019 Feb 3;22(3):475–84. doi: 10.1111/hex.12871 (PMC6543153; doi:10.1111/hex.12871)
Supplement: Supplementary file 1 [file HEX-22-475-s001.docx]

**Supplementary File**

**Definitions of screening, case-finding and diagnostic testing as given to the jurors.**

*Screening* – test all asymptomatic people in perhaps an age group or gender for dementia/cognitive impairment (eg. all people over 60 yrs)

*Case-finding* – a patient may incidentally complain about a problem (ie it is not the presenting problem) that triggers suspicion on the GPs behalf and so is tested for dementia (eg. complains of losing words 3 times this week, forgetting keys etc)

*Diagnostic testing* – a patient actively complains of symptoms suggestive of dementia and GP tests for dementia to diagnose.

**Community Juror recommended changes to the RACGP Clinical Guidelines for Preventive Activities in General Practice (section 5.5).**

In answer to the question “**Should the health system encourage GPs to practice “case-finding” of dementia in people older than 50?”,** the jurors noted that, in a perfect world, individuals over the age of 50 would not be subjected to routine case-finding of dementia. However, the jurors stated that, because case-finding currently exists, they wish to propose a number of caveats to current practices. These recommendations are as follows:

- No incentives to GPs for conducting case-finding of dementia.
- Current guidelines recommend monitoring and assessment of individuals over the age of 65; however, the jurors recommended the removal of this age bracket, instead offering all individuals equal access and equitable treatment regardless of age.
- Current criteria for classification of moderate risk of dementia includes individuals with symptoms, family history of Alzheimer’s disease, co-occurring medical conditions such as elevated cardiovascular risk, and low education. Jurors requested clarification of the last classifier, low education, to reflect not only education, but also degree of brain stimulation and activity.
- To complete case-finding and confirmation of a dementia diagnosis, jurors recommended that individuals be referred to specialists rather than have assessment and diagnosis completed by GPs, in an attempt to reduce over- and under-diagnosis, and misdiagnosis.
- Regarding current early intervention and prevention recommendations, the jurors suggested the inclusion of education regarding: the signs and symptoms of dementia; the processes of diagnosis; and, available treatment, support, and other services for individuals diagnosed with dementia. The jurors recommended that this education be provided to both GPs and the general population.
- Also regarding current early intervention and prevention recommendations, the jurors suggested an additional point. This point included the need for the Government to educate the community about lifestyle choices from an early age, for example in schools, not only for dementia, but also for other issues that may be affected in later life. This additional point was recommended in an attempt to minimise future health concerns.

**Post-CJ survey**

About your current attitudes towards case finding for Dementia

For each of the following, please circle a number from 1 to 7 on the scale that best describes how you feel now.

**For *you*, being tested for dementia is…..**

not beneficial 1 2 3 4 5 6 7 beneficial

unimportant 1 2 3 4 5 6 7 important

harmful 1 2 3 4 5 6 7 not harmful

not a good thing 1 2 3 4 5 6 7 a good thing

not worthwhile 1 2 3 4 5 6 7 worthwhile

About your current knowledge of dementia

(“correct answers” as recorded from expert presentations are highlighted and numerical information cited)

1. **The concept of case finding for dementia is best explained as when (circle one)**
2. a person has no symptoms of dementia and a doctor performs cognitive or physical tests to check for possible dementia
3. a doctor suspects that a person has dementia and performs cognitive or physical tests to check for possible symptoms
4. a person complains of known symptoms of dementia and the doctor performs cognitive or physical tests to check for possible dementia
5. **Case finding for dementia will detect all people going to the doctor that have dementia (circle one)**

**TRUE** **FALSE**

1. **Dementia is a neurocognitive disorder ranging from mild to severe (circle one)**

**TRUE** **FALSE**

1. **All cases of dementia progress to Alzheimer’s disease (circle one)**

**TRUE** **FALSE**

1. **There is little evidence that the medications such as cholinesterase inhibitors or statins affect progression to dementia (circle one)**

**TRUE** **FALSE**

1. **People over 70 years old, with low scores on cognitive tests performed by the GP will have dementia (circle one)**

**TRUE** **FALSE**

1. **Physical inactivity, diabetes, depression, and smoking place an individual at an increased risk of developing dementia (circle one)**

**TRUE** **FALSE**

1. **What proportion of people who have been diagnosed with mild cognitive impairment will progress to dementia? (circle one)**
2. 0%
3. 5-10 % (Mitchell & Shiri-Feshki, 2009)
4. 11-20%
5. 21-50%
6. More than 50%
7. **If we don’t screen or case find for dementia and a person is eventually diagnosed with dementia, how long is average delay before diagnosis?**

**(circle one)**

1. No delay, we find all dementia at case finding
2. Between 3 weeks and 6 months
3. Between 10 and 30 months (Savva & Arthur, 2015)
4. More than 30 months
5. **Of those who tested positive for dementia on case finding tests, how many people receive a diagnosis of dementia (true positive) when further testing is carried out?** **(circle one)**
6. Less than 20%
7. 21 – 40%
8. 41 - 60%
9. 60 – 70 % (Mate et al, 2017)
10. more than 70%

About Us

**Do you feel your opinions were respected by the group? (circle one)**

| 0 | 1 | 2 | 3 | 4 |
| --- | --- | --- | --- | --- |
| Not at all | A little | Somewhat | Reasonably | Very Much |

**Do you feel that the processes of the Community Jury were fair? (circle one)**

| 0 | 1 | 2 | 3 | 4 |
| --- | --- | --- | --- | --- |
| Not at all | A little | Somewhat | Reasonably | Very Much |

**How informed do *you* consider yourself to be about the harms and benefits of case finding for dementia? (circle one)**

| 0 | 1 | 2 | 3 | 4 |
| --- | --- | --- | --- | --- |
| Not at all | A little | Somewhat | Reasonably | Very Much |
